# How Do Home Care Workers Experience A Client's Death, Professionally and Personally? A Systematic Review and Meta-Synthesis

**DOI:** 10.1177/00302228241302431

**Published:** 2024-11-28

**Authors:** Gillian Mhairi Findlay, Noelle Robertson

**Affiliations:** 1 School of Psychology and Vision Sciences, 4488 University of Leicester, Leicester, UK

**Keywords:** death, grief, home health aides, caregivers, bereavement

## Abstract

**Background:** Home care workers (HCWs) may frequently experience client death. This critical interpretive meta-synthesis aimed to identify the impacts of client death to offer preliminary recommendations with respect to support. **Method:** Five electronic databases, APA PsycINFO, CINAHL, MEDLINE, Web of Science, and Scopus, were searched systematically using keywords and subject headings. **Results:** *Eight papers focusing on HCWs’ experiences of client death were identified*. *Constructs were elicited and interpreted via Reciprocal Translation Analysis*. *The interpreted line of argument highlighted four interrelated themes: ‘‘Personal grief: The impact of loss’, ‘Disenfranchised grief: no space for humanity and connection’', Inherent tension between self-care and organisational expectations: “You need a moment to hit the reset”, and ‘I wish someone was there to talk to: HCW support needs’*. **Conclusions:** The findings demonstrate that HCWs are affected in multiple ways by client death, but these impacts are not consistently recognised or responded to by their organisations.

## Introduction

Countries worldwide face population ageing and attendant challenges to meet an increased need for long-term care provision (hands-on assistance with activities of daily living over a substantial period; World Health Organisation [WHO], 2022). By 2050, one in five people will be 60 years or older, and the WHO recommends health and social care [HASC] service provision occur in local communities and people’s homes (2022). Professionals who provide such home care are variously known as home care workers [HCWs], home care aides, and home health aides. In this review, they will be referred to as HCWs. Irrespective of titles, core aspects of care provision are similar: helping older adults and individuals with disabilities and serious illnesses to stay in their homes by providing support around activities of daily living, mitigating risk in home environments, and offering limited healthcare support. This role extends beyond physical care to include relational and emotional elements, requiring that HCWs possess psycho-relational skills (Gazzaroli et al., 2020). Given the vulnerability of those being offered this home-based care, client death is not unusual. Whilst most deaths occur in hospitals, care homes, and hospices, over a quarter occur at home (28.7%, UK Government, 2023; 31.4% in USA; Olaisen, 2020). Given the privileging of home-based care, HCWs are likely to experience and be affected by client death.

Impacts of client death and dying on healthcare professionals have been documented in various clinical settings, with reviews on surgeons (Joliat et al., 2019), nurses (Bloomer et al., 2023; Guo & Zheng, 2019; Zheng et al., 2021), and physicians (Draper et al., 2019). All report lasting impacts on professionals, encompassing work and personal lives, particularly grief, defined as an emotional reaction to loss (Jacobsen & Petersen, 2020). Healthcare staff experiencing client death have also been subject to burnout (Maslach & Leiter, 2016) – a state of physical and emotional exhaustion from long-term stress, and compassion fatigue (Figley, 1995) - negative impacts on wellbeing and performance caused by frequent exposure to traumatic experiences of clients (Allie et al., 2018; Laor-Maayany et al., 2020; Slatten et al., 2020). The tenor of published literature has focused on assumed adverse experiences of client death. Less frequently, it has noted any enriching impacts, such as notably re-evaluating what is meaningful about life and its continuity (Sapeta et al., 2022; Sinclair, 2011). Indeed, in Roche et al.'s (2022) scoping review of 53 qualitative studies, which captured professionals’ experience of death in clinical settings, diverse emotional, physical, behavioural and spiritual impacts at both personal and professional levels were revealed, often affected by physical, cultural, economic, and regulatory contexts or environments.

Exploration of the impacts of client death on clinically-based professionals to date has meant domiciliary care workers, such as HCWs, are rarely included, and their work contexts are little examined. Yet key aspects of HCWs’ roles and working arrangements suggest a need to explore their experiences in-depth rather than generalise from research in clinical settings. HCWs deliver care within one of the lowest-paid, most insecure and precarious sections of the HASC workforce, with among the highest turnover rates (Turnpenny & Hussein, 2022). HCWs often lone-work and may have access to less organisational support than workers in residential settings (Assander et al., 2022; Hussein, 2018). Many roles require no formal qualifications, lack peer support, and are afforded insufficient training (Ravalier et al., 2019; Turnpenny & Hussein, 2022). Unsurprisingly, HCWs can experience stress arising from employment insecurity, low wages and status, and limited career progression (Bayley et al., 2023; Campbell et al., 2021; Mercille & O'Neill, 2020).

Impacts of client death may thus affect HCWs differently from HASC professionals working in inpatient or residential settings. For example, HCWs may lose income through a temporary reduction in hours until reallocation to a new client (Tsui et al., 2019). Client diversity (ages, abilities, health conditions and care needs) and variable exposure to clients receiving palliative care may also have impacts (UK Government, 2023). HCWs develop close relationships with their clients and families, which may render them vulnerable when death occurs. Grief has been reported as more intense when staff have close relationships, provide care longer, and feel emotionally unprepared; indeed, symptoms experienced by HCWs have been suggested as akin to those of family caregivers (Boerner et al., 2017). However, such grief is often not acknowledged or addressed, a phenomenon defined as disenfranchised grief (Doka, 1989, 2008).

To date, most published research examining the impacts of client death has focused on professionals in clinical or residential settings, or family caregivers. Studies utilising quantitative methods have primarily examined the frequency of exposure to death/dying or relationships between patient death and professionals’ symptoms of distress. Qualitative studies offering detailed insight into the complexity of HCWs' experiences of client death are emerging. Research has focused on specific issues, such as workers’ experiences of agency protocols (Barooah et al., 2019), coping strategies (Gee & Yates, 2021) and sources of support (Tsui, La Monica et al., 2021). However, literature related to how HCWs experience client death has not yet been subject to systematic review. Given the intrinsic and extrinsic stressors in terms of relational and occupational factors, knowledge of the detrimental impacts of client death on healthcare professionals in clinical contexts, and HCW’s increasing role in providing home-based care for an ageing population, it seems timely to understand experiences and impacts of client death on HCWs to better support and sustain this critical workforce. This review, therefore, sought to draw together published qualitative literature examining HCW’s experiences regarding client death to inform what is currently known about this, generate new knowledge, and identify directions for future research.

### Rationale and Aims

To date, research examining HCW’s experiences of client death has neither been systematically identified nor appraised or synthesised. However, parallel literature reveals that client death can cause grief and feelings of being unsupported and contribute to reduced job satisfaction, increased burnout, and turnover. Such effects may have a detrimental impact on health and wellbeing of both HCWs and their clients, as well as decrease retention of skilled caregivers.

The main aim of this review was to conduct an interpretive meta-synthesis of existing qualitative literature to provide a conceptual overview of how client death impacts HCWs to inform development of recommendations regarding support.

## Method

The qualitative meta-synthesis for this review followed a meta-ethnographic approach, as advanced by Noblit and Hare (1988). Meta-ethnography comprises a seven-phase, interpretive methodology for qualitative evidence synthesis, and it was selected since we sought to produce novel interpretations rather than aggregate existing findings. The former is achieved by systematically comparing conceptual data from included qualitative studies, facilitating the development of new concepts, theories, and models (France et al., 2019). Data for the synthesis process encompasses all information offered in findings/results and discussion sections (relevant to the research question) of all papers deemed to meet review inclusion criteria.

Our meta-synthesis drew on the following guidelines to ensure a robust review of the literature: eMERGe guidance for the reporting of meta-ethnography (France et al., 2019), Preferred Reporting Items for Systematic Reviews and Meta-Analyses’ [PRISMA] (Page et al., 2021), and CASP Qualitative Studies Checklist (CASP, 2018). The review was registered on PROSPERO.

### Information Sources and Search Strategy

A search strategy was developed using the SPIDER acronym (Table 1; Amir-Behghadami & Janati, 2020).

**Table 1.** Search Strategy.

Sample:	Individuals who provide personal care in a paid employee role to clients in their own homes
Phenomenon of interest:	Client death
Design:	Qualitative research studies
Evaluation:	“Impact” OR “effect*” OR “experienc*” OR “attitude*”
Research type:	Qualitative OR mixed methods

Qualitative methodologies were chosen given their scope to offer nuanced data to understand impacts on HCWs, with meta-synthesis offering a systematic approach to searching, synthesising, and reinterpreting data that presents detailed descriptive findings (France et al., 2019; Lachal et al., 2017; Sandelowski et al., 1997).

Initial scoping searches were conducted by the primary author using Google Scholar and APA PsycINFO to identify relevant papers. The search term strategy focused on four search strings based on ‘job role’, ‘bereavement’, ‘methodology’ and ‘impact’. The search was conducted on APA PsycINFO, CINAHL, MEDLINE, Web of Science, and Scopus in June 2024. Initial results were refined using search limiters. Reference lists were examined to identify other relevant studies.

### Eligibility Criteria

For the synthesis, peer-reviewed qualitative studies published in English and reporting the experiential impact of client death on HCWs were included. Studies were excluded if they focused solely on client, family, or unpaid caregiver experiences or if participants provided home-based care within a role other than HCW (such as a qualified nurse). Since we only sought qualitative or mixed methods; papers were also excluded if data for HCWs were not discernible. Editorials and reviews were excluded as they did not offer new data. No time limit was applied to searches. See Figure 1 for details.

**Figure 1.**
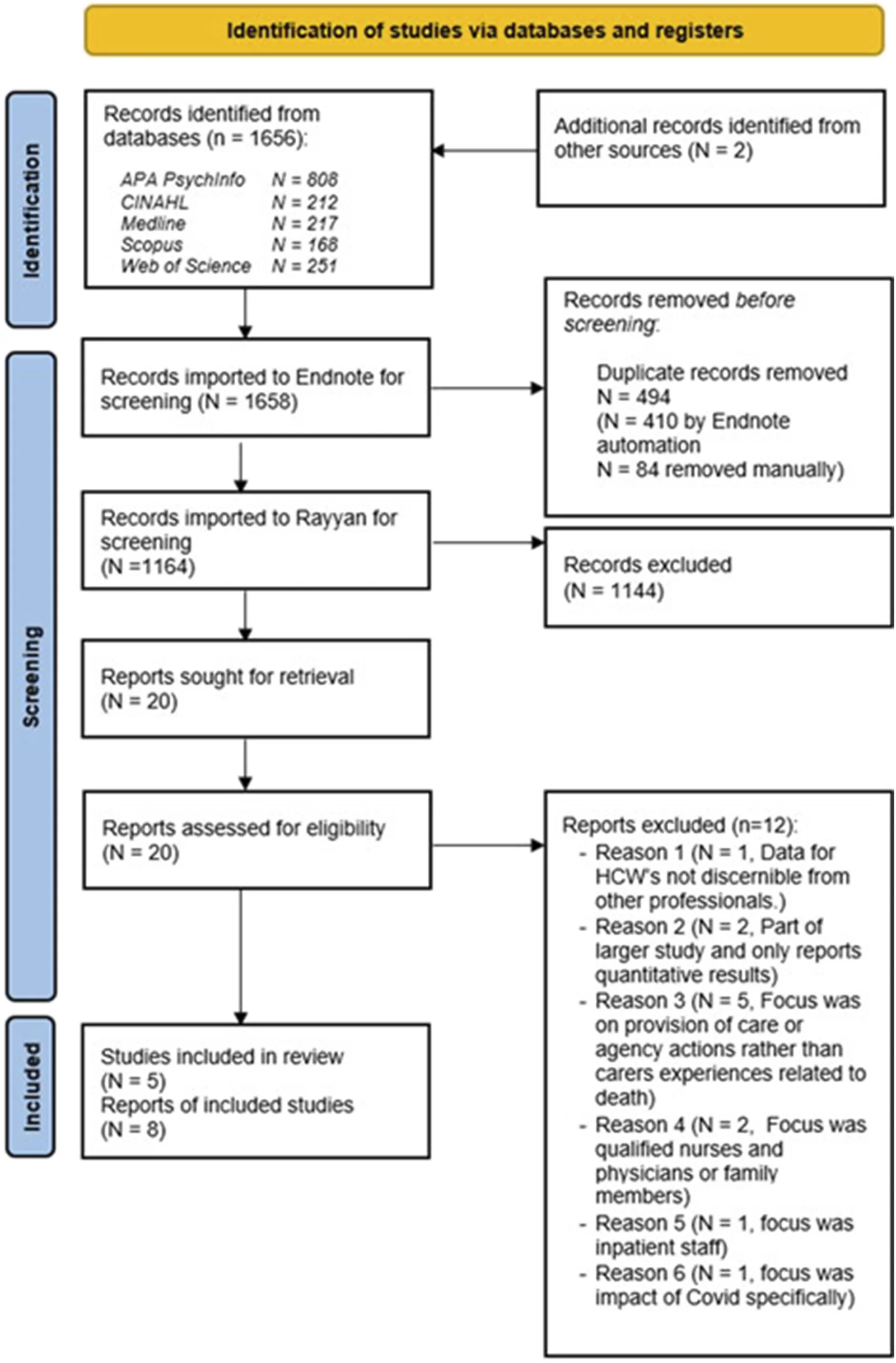
PRISMA (Adapted from Page et al., 2021).

### Selection Process

The initial database search identified 1164 unique papers once duplicates were removed, including two papers identified from manually searching reference lists from key studies. The authors initially screened all titles and abstracts by applying the following criteria: (1) Does the study focus on client death? and (2) Does the study explore HCWs’ experiences of client death? From this, 1144 papers were excluded; 20 qualified for full-text review either because they met inclusion criteria or could not be excluded by the title and abstract alone. At full-text screening, 12 further papers were excluded (see Figure 1), resulting in eight papers for quality assessment. These eight papers reported results from five studies (one reported across three papers and another across two; Figure 1).

### Assessment of Methodological Quality of Included Studies

Debate continues about assessing methodological quality and rigour in qualitative research and suggests that methodology and conceptual frameworks may have more relevance than quality appraisal when synthesising qualitative studies (Atkins et al., 2008). However, the inclusion of poor-quality research may prompt questioning regarding the integrity of findings (Toye et al., 2013). In this review, we opted to undertake explicit quality appraisal to identify study limitations which may have influenced results of this review and inform appropriate weighting for the current meta-synthesis.

The CASP (CASP, 2018) qualitative checklist was used to assess quality of included studies. This checklist considers the validity of results (appropriateness of design), results (data analysis, consideration of ethical issues and clarity of findings) and value of results. A rating system was used, whereby a score of half, one or zero points was assigned depending on whether answers to each question were ‘partially’, ‘yes' or ‘no', respectively. Each study was reviewed against CASP by the first author, with the second author assessing four included studies, reaching an inter-rater reliability statistic of 1 (Cohen’s Kappa; McHugh, 2012). Eight studies were included in the final analysis (Table 2).

**Table 2.** Study Profiles.

Source Paper (*n* = 8) &Country	Barooah et al. (2019),^a^ USA	Boerner et al. (2016),^a^ USA	Gee and Yates (2021), UK	Ghesquiere and Bagaajav (2020), USA	Gleason et al. (2016),^a^ USA	Tsui et al. (2019), USA	Tsui, LaMonica et al. (2021) USA^b^	Tsui, Reckrey, et al. (2022), USA^b^
Research aims	To understand how HHAs experience agency’s practices immediately after client death	To compare HHA experiences between two agencies with clear positions regarding follow-up after patient death	To explore experiences/coping strategies of homecare workers following patient death	To understand HHAs’ reactions /Coping following patient death	To explore supervisor and co-worker support among HHAs, leading up to client’s death and after	To explore mechanisms that disenfranchise homecare workers’ grief	To explore how aides make use of sources of support to navigate client death, and what problems arise	To describe end-of-life care experiences of aides providing a mix of home-based hospice and non-hospice care
Theoretical framework	Thematic	Thematic	Interpretive phenomenology	Thematic	Thematic	Thematic	Thematic	Thematic
Sample Gender: Age: Experience:	Purposive HHAs (*n* = 80) 96% female 24–69yrs (*M* = 43.2) 16–29yrs (*M* = 6.5)	Purposive HHAs (*n* = 78) 96% female 24–69yrs (*M* = 43.2) 16–29yrs (*M* = 6.5)	Purposive Home carers (*n* = 6) 100% female 29–64yrs (*M* = 43.2) 4–33yrs (*M* = *NR*)	Purposive HHAs (*n* = 14) *NR* *NR* *NR*	Purposive HHAs (*n* = 80) 96% female 24–69yrs (*M* = 43.2) 16–29yrs (*M* = 6.5)	Purposive Homecare aides (*n* = 26) 100% female 32–60yrs (*M* = 43.2) 1–25yrs (*M* = 10)	Purposive Homecare aides (*n* = 29) 100% female 30–67yrs (*M* = 53) 1–27yrs (*Md* = 8)	Purposive Homecare aides (*n* = 29) 00% female 30–67yrs (*M* = 53) 1–27yrs (*Md* = 8)
Race/ethnicity	Black (67%), white (10%), Asian/Pacific Islander/native (4%), American (4%), other (19%) Hispanic (29%)	Black (67%), white (10%), Asian/Pacific Islander/Native (4%), American (4%), other (19%) Hispanic (29%)	*NR*	*NR*	Black (67%), white (10%), Asian/Pacific Islander/native (4%), American (4%), other (19%) Hispanic (29%)	*NR*	Black (6%), mixed (14%), white (14%), Asian (7%) Hispanic (20%)	Black (6%), mixed (14%), white (14%), Asian (7%) Hispanic (20%)
Methodology	Semi-structured interviews	Semi-structured interviews	Semi-structured interviews	Focus groups and semi-structured interviews	Semi-structured interviews	Qualitative focus group and peer interviews	Semi-structured interviews	Semi-structured interviews
Analysis	Qualitative analytic analysis	Qualitative analytic analysis	Interpretive phenomenological analysis	Thematic analysis	Thematic analysis	Thematic analysis	Thematic analysis	Thematic analysis & framework method
Ethical considerations	Institutional approval and informed consent	Informed consent	Ethical approval	Institutional approval	Institutional approval and informed consent	Institutional approval and informed consent	Ethical and institutional approval, and informed consent	*NR*
Reflexivity described	Reflective group discussions	Recruitment rationale reported	Reflective group discussions	*NR*	*NR*	Reflexivity, triangulation, and peer debriefing	Reflexivity, triangulation, and peer debriefing	Reflexivity, triangulation, and peer debriefing
Quality appraisal rating	7.5	7.5	8.5	8.5	7	8.5	9	8

^a^Papers derived from same primary study but a different focus. Participant demographics stated the same in both papers, although 2 participants were excluded in Boerner et al. (2016).

^b^Papers derived from same primary study/same participants but different focus.

*NR* Not reported.

### Interpretive Meta-Synthesis

Meta-ethnography is a process that views individuals’ narratives as their interpretations of their experiences (Noblit & Hare, 1988). Researchers’ experiences can also influence interpretation of data; thus, a conceptual distinction has been proposed between first-order constructs (individuals' own narratives) and second-order constructs (researchers’ interpretations of these narratives). Meta-synthesis combines multiple studies to create third-order constructs through interpretations of interpretations, considering the contextual and methodological complexities of each study (Zimmer, 2006).

All relevant data from studies, including basic data about study designs, settings, samples, participant quotes and author comments, were extracted for this review (Table 3). France et al. (2019) highlight challenges in distinguishing between first and second-order constructs due to authors selecting participant quotes to support their second-order constructs. Therefore, all data were extracted with original wording being preserved, and first-order constructs were analysed and synthesised along with their corresponding second-order constructs, not in isolation (France et al., 2019).

**Table 3.** Third-order Constructs.

*“Personal Grief”: The impact of loss*
• HCW responses following client death • Nature of relationship impacts grief • Role of acceptance
*Disenfranchised Grief: No space for humanity and connection*
• Emotional impacts compounded by lack of recognition • Organisational silence regarding grief • Lack of shared humanity
*Inherent tension between self-care and organisational expectations: “We need a moment to hit the reset”*
• Tensions with work preferences during grieving • Challenges implementing strategies to safeguard own wellbeing • Challenges managing ongoing contact with families
“*I wish someone was there to talk to*”: *HCW support needs*
• Experiences of support from agencies • Support desired from agencies

We used an iterative process to note and discuss ideas and additional interpretations to develop third-order constructs. Themes were consistent across the papers, allowing translation of the studies while maintaining the complexity of reported experiences. Extracted themes were developed alongside repeated reference to original texts to ensure meaning was not lost, and all core themes captured (Zimmer, 2006). Interpretations were refined to comprise four interconnected themes, derived through combining similarities and identifying difference across papers. Across the eight papers there appeared to be a largely consistent argument regarding what HCWs’ experiences of client death are (Noblit & Hare, 1988). Even when concepts were not identified in a particular paper, the relationship between papers was still deemed to be reciprocal. Differences were identified during this process; however, concepts were not contradictory; they instead described different perspectives of the same phenomenon. A line of argument synthesis was therefore considered most appropriate for the aim of this synthesis, drawing on the approach of Sattar et al. (2021). The findings are discussed under the theme headings of impact of loss, disenfranchised grief, caring for self, and identified needs (Table 3).

## Results

### Description of Studies

The eight identified papers were published between 2016 and 2022 in the USA (seven) and UK (one) (Table 3). Results were reported from five studies, including data from 155 HCWs who had experienced client death. The mean sample age was 46 (range = 6–80 years). Data were collected through semi-structured interviews for all studies, with one study employing a method where they trained participants to undertake peer interviews with other participants and two studies utilising focus groups.

## Findings

This section comprises HCWs’ quotes and author commentaries from the eight identified papers. Thematic diagrams depicting the categories, themes, and sub-themes are included in Figures 2, 3, 4, 5.

**Figure 2.**
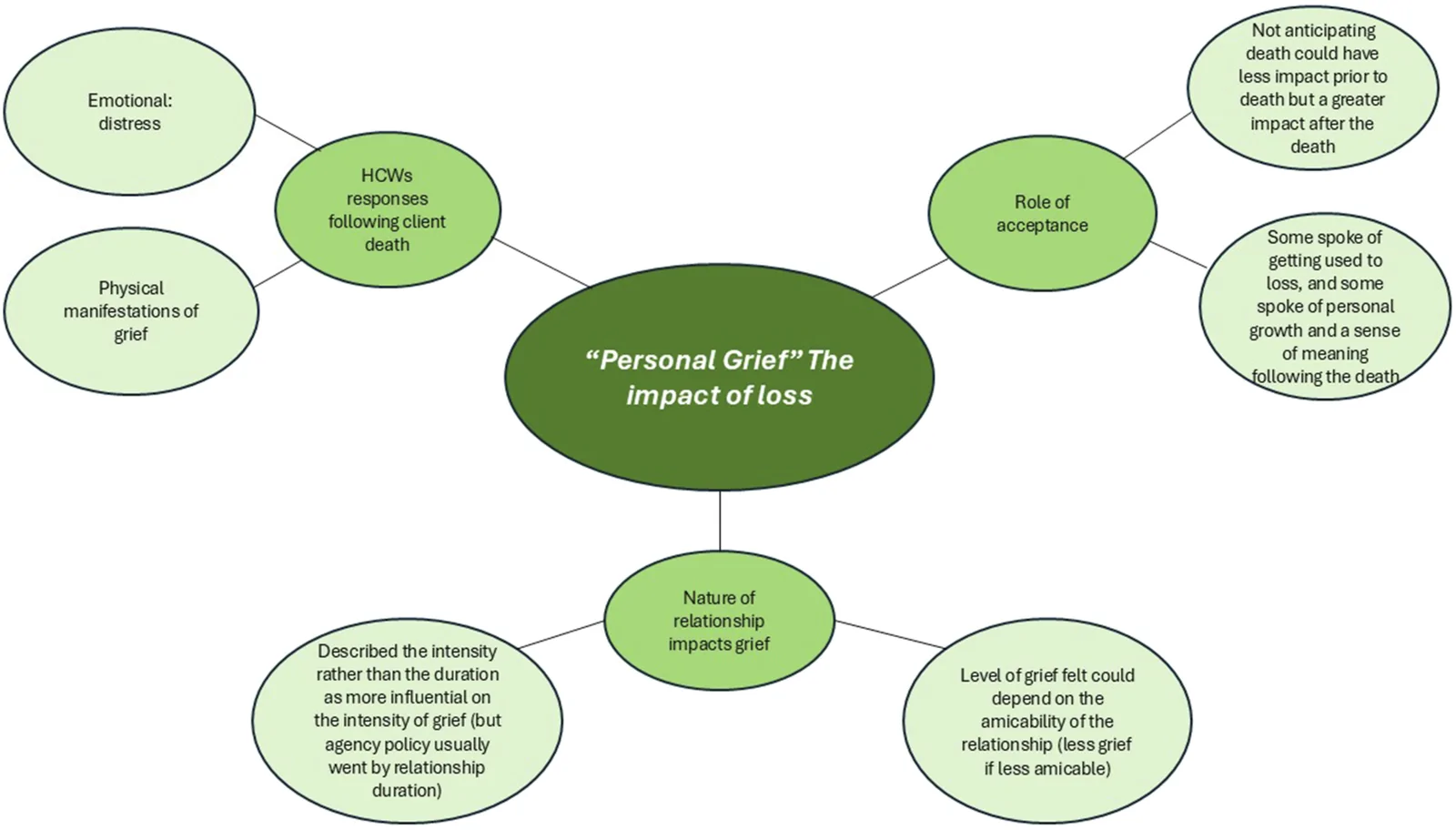
Personal grief experienced by HCWs following the death of a client.

**Figure 3.**
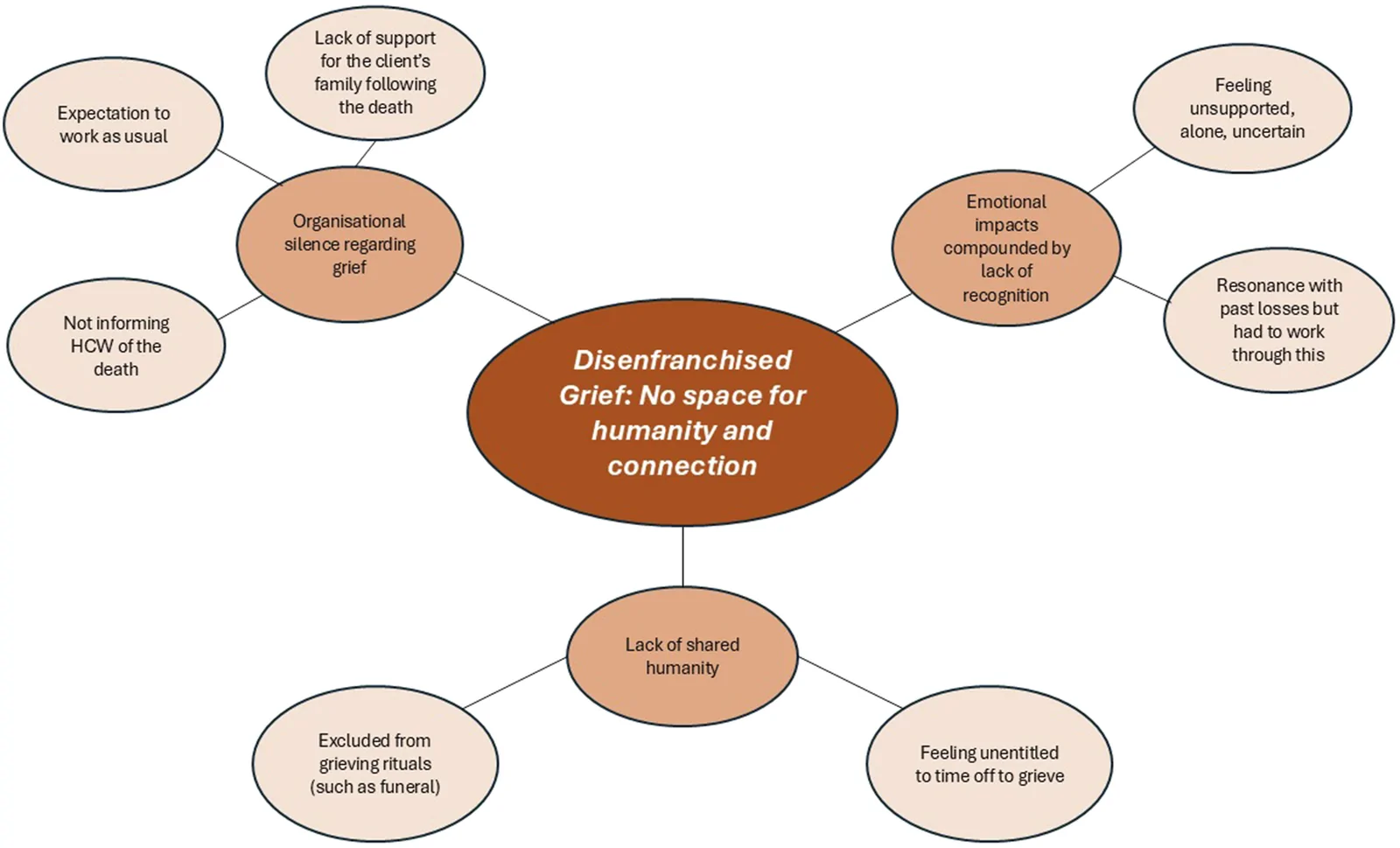
Disenfranchised grief.

**Figure 4.**
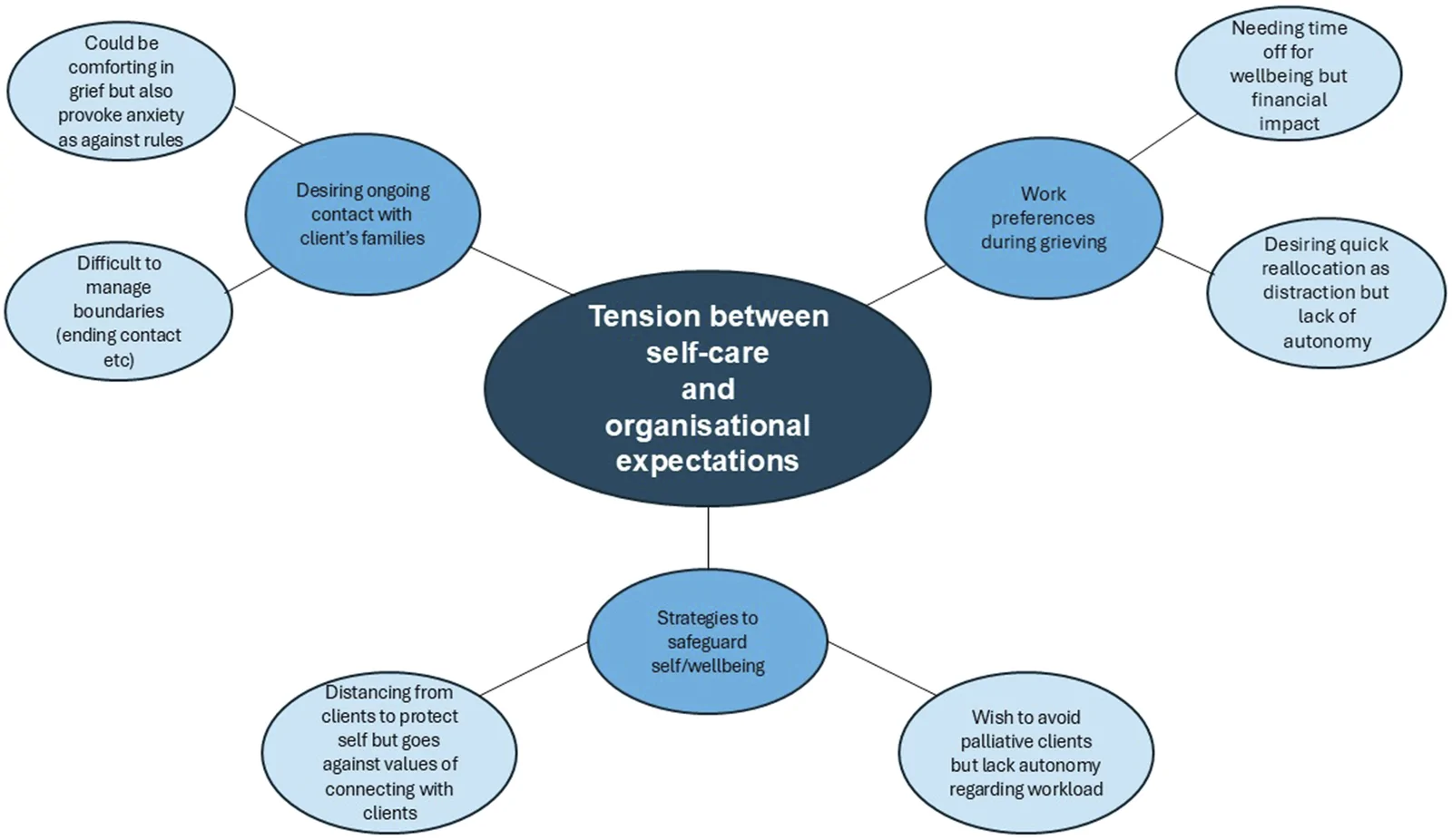
Tension between self-care and organisational expectaions.

**Figure 5.**
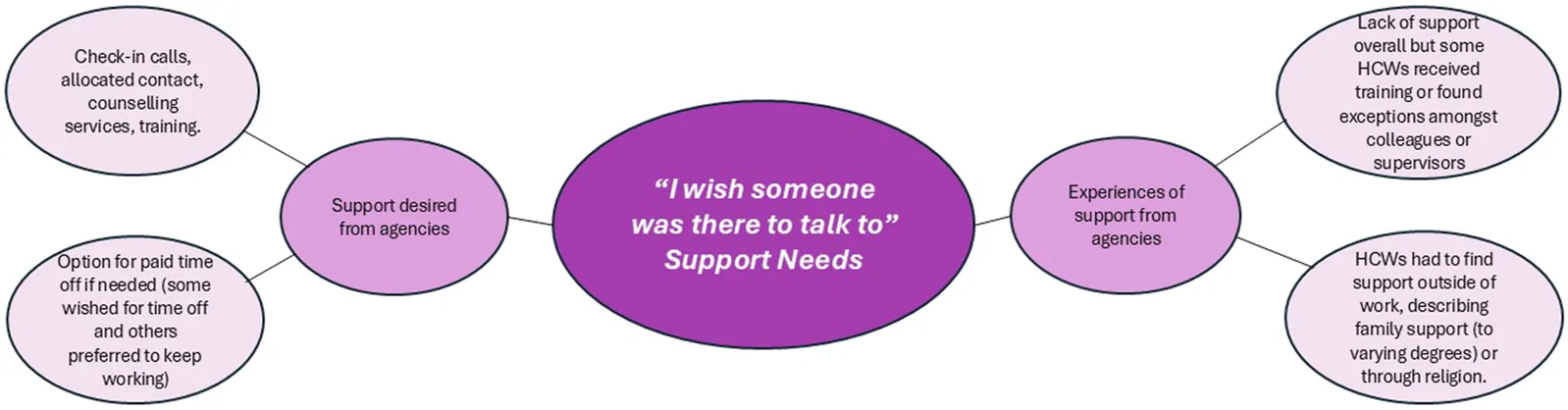
Experiences of support and desired support.

### *“*Personal Grief”: The Impact of Loss

HCWs experienced “personal grief” (Gee & Yates, 2021, p. 248) following the client’s death, which manifested both emotionally and physically, irrespective of how long the care relationship had existed. Its extent appeared related to the emotional intensity of relationships rather than duration.

HCWs described diverse and, at times, overwhelming responses to client's death, *“It [affected] me in a way that I never realised it would*.*”* (Tsui et al., 2019, p. 385). Some HCWs experienced *“an outpouring of grief at home”* (Gee & Yates, 2021, p. 250) and described, “*Every death is like something is missing from you…*” (Gee & Yates, 2021, p. 249). HCWs outlined emotional impacts: distress, sadness, anger, shock, numbness, grief, relief, confusion, guilt, lack of desire to socialise, and feelings of emptiness. They also experienced physical symptoms: exhaustion, headaches, loss of appetite, increased blood pressure and sleeplessness. For some HCWs, guilt, coupled with anxieties about future deaths, led to time off work, *“I'm feeling so guilty*. . . *I didn't want to come back to work*.*”* (Tsui et al., 2019, p. 385).

One HCW described “*not being able to accept the client's death ‘took a mental toll*.'” (Tsui et al., 2019, p. 385), whereas another highlighted a need to accept it was “*their time and they have to go*” (Tsui, Franzosa, et al., 2022, p. 7). Tsui, Reckrey, et al. (2022) proposed those “*in denial or not aware of a case's [End of Life] status may experience less strain during the life of the client but could experience more grief afterwards due to reduced preparedness for death and a sense that they did not do enough*” (p. 12). Some HCWs reported they became more used to losses over time, whilst others feared a “*level of acceptance made them feel emotionally shut down, and that it could be difficult to express emotions about other things in their lives”* (Ghesquiere & Bagaajav, 2020, p. 621).

The nature of relationships “*shapes an aide's experience of death*” (Tsui et al., 2019, p. 385). HCWs spoke about “*caring as more than a job*” and “*such investment also increased carers' vulnerability to grief*.” (Gee & Yates, 2021, p. 249). This investment appeared to underpin HCWs describing strong emotional reactions after caring for someone for “*just three weeks […] If you put your heart in what you are doing, you will feel it*” (Gee & Yates, 2021, p. 249). However, some HCWs described less acute feelings of grief where relationships were less emotionally rewarding or were fraught. One HCW described the loss of a client who was “*never pleased” and “calling you all [kinds of names]”* as *“less emotional*” (Tsui et al., 2019, p. 385). HCWs experienced agencies as willing to bend the rules (which discouraged close client relationships and prevented funeral attendance) based primarily on the duration a HCW had supported a client. Agencies did not appear to recognise that short relationships could evoke strong emotional reactions.

For some respondents, client death offered possibilities for growth and *“a sense of meaning and satisfaction with their role”* (Tsui et al., 2019, p. 386); it could raise “*existential questions*” (Gee & Yates, 2021, p. 252), “*It brings me to a point of reflection … and reminds me how important life is and the value of life, having family, friends and loved ones*.” (Gee & Yates, 2021, p. 251).

### Disenfranchised Grief: No Space for Humanity and Connection

HCWs described their grief as not recognised or acknowledged, *“she was a living human being*. *[…] ‘Don't go back, she passed'- a little heartless”* (Barooah et al., 2019, p. 20). This 'disenfranchisement' of grief (Doka, 1989, 2008) affected them in different ways.

Some HCWs reported shock at client loss since they were not explicitly notified by their agency, discovering only incidentally in the community or through contact with clients' families, *“One of the grandchildren at my church said, “Did you know Grandma passed?” … [family] wanted us to come to the funeral, but [agency] never gave us information*.*”* (Barooah et al., 2019, p. 21). Some only found out when arriving at clients' homes, with an additional stressor of being responsible for notifying their agency. Other HCWs spoke about rules precluding HCWs from being notified but were grateful individual coordinators showed flexibility, *“I was glad that they told me because they aren't really supposed to*.*”* (Barooah et al., 2019, p. 21).

Lack of recognition as a grieving party by employers and their family networks could contribute to HCWs feeling alone, unsupported, and uncertain of their roles or how to respond. The emotional impact of client loss could also resonate with previous losses. Gee and Yates (2021) describe, *“Like a gas leak, carers' memories of personal loss seeped into their work with their patients, corroding the space that separated the grief for the patient from their own personal losses*.*” (p*. *251)*. HCWs identified with clients and families, making links to their own experiences, or imagining experiencing the same loss, *“I had just been through a death myself…I felt like it, he's also like my father who is dying, I couldn't cope” (*Gee & Yates, 2021*, p*. *249)*.

The failure to recognise grief appeared allied to deficient organisational responses, notably expectations that they should continue working as usual, irrespective of a client dying. HCWs reported little opportunity afforded them to process their feelings and often felt they must *“try to temporarily set aside their grief so that they could focus on their work”* (Ghesquiere & Bagaajav, 2020, p. 622). Some HCWs did not feel entitled to time-off or support even when struggling. HCWs felt they were not treated as humans, exemplifying a lack of provision for time to grieve between a client dying and being allocated to a new client, *“…an hour after she died, they were giving me another case! They treat me like I was a number, that's it - not a human being”* (Barooah et al., 2019, p. 23). However, some HCWs preferred quick client reassignment, *“It was like relief and a distraction to the mind”* (Barooah et al., 2019, p. 22).

Tsui et al. described the bind of HCWs as being “*doubly disenfranchised: When clients die, aides are wrongly perceived as having both insufficient losses and insufficient relationships to justify their grief and merit support*.” (2019, p. 389). Finally, not being recognised as a grieving party (and having no formal recognition within agency protocols) often precluded participation in rituals which could support individuals with grief, like funerals or contact with clients’ families.

### Inherent Tension Between Self-care and Organisational Expectations: “You Need a Moment to Hit the Reset”

HCWs described both immediate and longer-term effects of client loss and different ways in which they tried to cope and care for themselves, reported as challenging in a working context where roles lacked autonomy and supportive benefits (such as paid time-off). Self-care had to be traded against the need to meet employer requirements and avoid risking job loss. Participants described taking time-off and utilising coping strategies such as keeping busy and distraction and identifying ways to safeguard their future wellbeing (such as wishing to avoid end-of-life care assignments to reduce exposure to loss). Another factor in this complex balancing act was their values and desire to provide high-quality patient care within a culture where human connection and relationships were not valued and emotional labour was not recognised. HCWs could try to remain distanced from clients to protect themselves from further emotional distress, but this conflicted with their core values.

HCWs, across all studies, described “*an inherent tension in balancing financial security and emotional health*” (Tsui et al., 2019, p. 386). Many recognised they needed time-off due to the emotional impact of loss, but there were adverse financial consequences, *“…in a way I took my mourning days but the agency does not pay […] After a week I had to come back because of the bills”* (Barooah et al., 2019, p. 23). Some HCWs wished for rapid client reallocation (for financial and emotional reasons) and struggled when agencies could not facilitate this. HCWs who had to take on a new client when they did not feel emotionally ready “*appear to have the greatest need for additional supports*” (Tsui et al., 2019, p. 388). One participant regretted her decision to take on a new client immediately, “*I started working right away, trying to, you know, not focus on it*. *And it was too soon…*” (Tsui et al., 2019, p. 387).

Being pushed to continue end-of-life care work could negatively impact HCWs’ wellbeing (Tsui et al., 2019). “*I didn’t wanna do hospice ever again in life because it was a feeling [that] I never ever wanna feel again*” (Tsui, Reckrey, et al., 2022, p. 5). Some HCWs wished to avoid end-of-life care assignments to reduce exposure to loss but continued to be assigned these or remained off work to avoid these. There was evident tension for this professional group in prioritising self-care with financial security, with negative impacts of client loss exacerbated by this pressure and lack of autonomy in their role. Tsui et al. summarised: “*emotional effects can amplify the financial effects of a client death, making aides’ experience of grief more dynamic and intricate than others may easily see or understand*.” (2019, p. 389).

When HCWs take time off due to grief, *“they often see it as their choice to go unpaid, despite the need for time-off stemming from the work*.*” (*Tsui, Reckrey, et al., 2022*, p*. *8)*, and could perceive it as an active choice to lose income, *“No, I didn't get support … I just took my days off*. *I chose to lose the pay … because death is real and it's fragile … you need a moment to hit the reset”* (Tsui, La Monica et al., 2021, p. 367).

For HCWs across all studies, there were frequent tensions between preferred coping strategies and professional and personal values. For example, some HCWs tried to maintain boundaries to prevent themselves getting too close to clients, “*You have to defend yourself, you have to not let yourself get too close … because they [the agency staff] tell you not to*. *And if you don't want to hurt yourself in that way, try to find ways not to get very close to them*.” (Tsui et al., 2019, p. 387). Yet, keeping emotional distance, which did not align with their values of providing quality care and connecting with clients, “*If it doesn't come from inside, you will not enjoy it*” (Gee & Yates, 2021, p. 249) was understood as an unresolved dilemma.

Many HCWs desired continued contact with clients' families, from which they drew comfort: “*The family had a little get-together and they invited us … So that was a thing in terms of coping*” (Tsui, La Monica et al., 2021, p. 367). HCWs reported small tokens as being powerful in feeling recognised and valued, “one participant described how a client's sister advocated for her, telling her: “*I'm so thankful for what you did for my sister*. *… She did call my supervisor and told her, you know, give me the best job she has*” (Tsui et al., 2019, p. 386). Contact was often short-term, but some HCWs sustained this longer term, “*…I think if I hadn't been in touch with them, it would've felt sad and lost*. *… I would've taken it on more*” (Tsui, La Monica et al., 2021, p. 367). However, sustained contact came with the risk of attachment and more contact than the HCW could provide, such as frequent visits or phone calls. Agency policy often precluded ongoing contact; however, HCWs sometimes chose to breach this policy but could experience anxiety about breaking the rules.

### “I Wish Someone was There to Talk to”: Support Needs

HCWs spoke of the necessity of support, “*…you definitely need somebody to talk to, someone who you can confide in*. *[…] You can't do this alone*.” (Tsui et al., 2019, p. 387). A lack of support from agencies prompted HCWs to rely on personal strength or family members*, “You know, friends and family are always there for support*. *But basically, that's it”* (Tsui, Franzosa, et al., 2022, p. 367). Indeed, family members with similar work roles could offer a particular type of shared understanding, “*I do have an aunt who's a nurse and she's experienced death on many occasions, so that's very helpful*.” (Tsui, La Monica et al., 2021, p. 367). However, some HCWs spoke of family members not understanding, *“Support with me wasn't in my family cuz they don't care* (Tsui, La Monica et al., 2021, p. 367).

Some found religious support to be helpful, “*The spiritual approach is that there is always help available*. *Personally, there's a prayer hotline that can be used when I can't go within myself to do it [e*.*g*.*, cope with client death]”* (Tsui, La Monica et al., 2021, p. 367).

Some participants spoke of other HCWs providing support, *“We see other hospice workers*... *[and they]* ... *will say ‘Oh, I’m sorry to hear that your patient died,’ and we will talk and* ... *we will hug each other*.*’’* (Ghesquiere & Bagaajav, 2020, p621). This informal support was valued, especially given the lack of formal support structures in employing agencies. However, supportive peer relationships were not always present and even when established, they did not always last, “*because we all have our different problems and cases*. *You don’t have enough time”* (Tsui, La Monica et al., 2021, p366).

Across studies, HCWs identified a range of ways they felt employing agencies could better support staff, notably recognising the caring relationship and the HCW’s loss; considerate death notification; a check-in from the supervisor; paid time-off or quick reallocation, depending on the HCW’s preferences; counselling/support groups; contact with the client’s family; and training around end-of-life care, death/dying, and working with family members.

## Discussion

A meta-ethnographic approach was used to synthesise published qualitative literature exploring HCW’s experiences of client death. The review of eight studies revealed negative impacts on HCWs and how contextual factors, such as work role, status, economic situation, and lack of organisational support, may increase vulnerability to adverse psychological outcomes. The interpreted line of argument highlighted four broad, interrelated themes: impact of loss, disenfranchised grief, tension between self-care and meeting organisational expectations, and HCW’s support needs. Consistently across papers, *“the responsibility of enfranchising grief fell solely on individual home care workers who struggled to cope with the emotional effects of client death while trying to make the most optimal financial decisions possible”* (Tsui et al., 2019, p. 389)

HCWs experienced personal grief following client death, describing a range of related emotions and physical symptoms, echoing previous research around the impacts of client death on HASC professionals (Bloomer et al., 2023; Draper et al., 2019; Guo & Zheng, 2019; Joliat et al., 2019; Zheng et al., 2021). Given multiple and well-established models of grief which highlight the psychological processes people experience (e.g. Stroebe and Schut’s (1999) dual-process model and Kübler-Ross and Kessler’s (2005) sequential stage approach, it is perhaps surprising that participants in this review were not afforded the opportunity to focus on their loss or to reorient to their world, with their client gone. The critical processes of meaning-making, shifting biopsychosocial demands, and altering relationships between the living and the deceased are highlighted in contemporary psychological understandings of grief (Silverman et al., 2020). Yet, home care organisations appear to be failing to recognise the necessity of these processes, with staff experiencing distress in consequence.

That relationship intensity rather than duration influenced grief experienced for some HCWs was of note. Whilst this is noted in previous literature (Tang & Xiang, 2021), contrastingly, longer care relationships can increase grief intensity in staff, and the longevity of relationships is often assumed to be key in grief intensity and intractability (Boerner et al., 2015). Complex relational care around clients’ emotional, social, and cognitive wellbeing is frequently an intrinsic part of HCWs' roles (Franzosa et al., 2018; Reckrey et al., 2019), and it may be that HCW relationships with clients develop quickly due to working in their homes and being the primary care providers. This finding highlights the importance of assessing individual responses to client death rather than assuming a minor impact after relatively brief working relationships.

HCWs also described disenfranchised grief whilst taking individual responsibility for wellbeing within a role offering minimal autonomy; this seems to sustain tension and dilemmas, increasing pressure for staff already vulnerable after client loss. Yet recognising grief reactions and assessing staff’s experience with death is argued to be critical for delivering compassionate care. Validation of feelings of loss can mitigate distress and foster personal growth (Gilbert et al., 2021), so there are compelling reasons for organisations to support employees through experiences of loss and bereavement. Lack of support can exacerbate complex grief reactions (Pop-Jordanova, 2021). Indeed, healthcare staff who cope with death less effectively risk delivering poorer future care due to reduced work performance (Mir et al., 2024), which, evidenced in palliative care settings, can contribute to burnout, compassion fatigue, and poor retention (Hussain, 2021). Employers should offer a compassionate and nuanced assessment of the impact of client death to enable HCW time and agency to process loss to reduce staff distress and support optimal care delivery.

Data within this review (from North America and the UK) arguably emerges from neoliberal societies where care is outsourced, care roles devalued, and care staff marginalised in terms of agency, rank and gender (Banks, 2018; Mancha et al., 2022; Scales, 2021). HCWs had to trade-off self-care against organisational expectations. A lack of power and status may contribute to HCWs internalising feelings of low worth and low esteem (Banks, 2018; Mancha et al., 2022) and feeling disempowered to challenge their employer’s lack of recognition of the relational nature of the work and the bereavement. HCWs may also experience pressure to employ increased ‘emotional labour' (managing the dissonance between the level of personal feelings and professional expectations of behaviour; Hochschild, 2012) when supporting family members. This may often be in a context where they are unable to request involvement from their agencies (due to policies prohibiting contact). Within long-term care facilities, staff members’ beliefs that emotional distance is expected appeared to reinforce organisational cultures of denial and silence around death and dying (Ramvi et al., 2021). Banks (2018) highlights the absence of care and support across systemic levels in aged care (community to care sector, policy to care sector and care sector to employees and clients) as a potential source of 'moral injury’ (where strong cognitive and emotional responses follow violations of an individual’s moral code, potentially causing distress (Williamson et al., 2021). Recognising the broader systemic challenges within the care industry may offer some explanation for the embedded cultures; however, there appears to be an evident need for change.

### Recommendations

A key emergent theme in this review privileges the importance of organisational management in response to client loss. HCWs identified numerous ways in which employing agencies could better support staff, notably recognising the caring relationship and HCW’s loss; considerately informing of client death; a check-in from supervisor; paid time-off or quick reallocation, depending on preferences; counselling/support groups; contact with client’s family; and training on end-of-life care, death and dying, and working with family members. This type and extent of response to client death are likely to require cultural awareness and understanding, acknowledgement and planning for normal emotional reactions to situations such as bereavement. A culture change, underpinned by regular team communication and training on loss and grief, may also enable HCWs to recognise grief and enter discussions about their needs. Issues relating to ongoing agency support, knowledge of bereavement support policies/resources (including time-off) and uncertainty about levels of permitted contact or involvement in grieving rituals were evident. The absence of organisations’ communication with staff regarding grief was evident across all studies, with no apparent sense that agencies understood the impacts or their complexities on staff. HCWs linked organisational deficiencies to a need for more individualised planning or accommodation alongside up-to-date policies and procedures and sharing knowledge regarding normal grief reactions.

Adequate employee bereavement support could extend beyond an initial period of bereavement leave. HCWs spoke of individual preferences to have time-off between clients or quick reallocation (if preferred and with consideration of whether the motives are linked to emotional coping or financial) and allocation to different client groups (such as clients not receiving palliative care). Within a short-staffed service, it is recognised that there may be challenges, but collaborative discussion and problem-solving with HCWs may make a significant difference in their experiences and offer a greater sense of autonomy. When considering HCWs’ contact with family members or participation in rituals, HCWs need clear guidance on what is or is not permitted and what flexibility can be applied, which could contribute to a more supportive and understanding work environment.

### Strengths, Limitations and Reflexivity

A comprehensive search strategy was implemented across reputable databases to ensure the selection of articles relevant to the research’s aims. However, it is important to acknowledge that despite our best efforts, it is possible that some relevant papers may not have been captured by the search strategy.

The review’s included studies are relatively recent and from only two countries, reporting on relatively small, predominantly self-selecting samples. HCW demographics and employment contexts do differ. In England, the majority of HCWs are female (82%), white (74%) and British (79%) (Skills for care, 2023). In the USA, the majority of HCWs are women (87%); however, over 62% come from racialised backgrounds, and 31% are immigrants (Campbell et al., 2021). The potential for marginalisation and disempowerment of HCWs must be considered (see Hussein, 2022) and is possibly more salient in the USA. Reliance on a private healthcare insurance system will likely also shape American HCWs’ experiences differently (particularly regarding financial precarity). However, in both countries, core tasks are similar, as are low pay, low status, and constrained opportunities for professional advancement (Campbell et al., 2021; Skills for care, 2023). Therefore, the results of this synthesis are considered transferrable to the UK context, where minimal research has been completed on this topic.

Only English-language studies were included, which may account for the lack of papers from other countries. This is an important consideration for future research, as the structure of home care services and salient cultural attitudes to death and dying are likely to influence HCWs’ experiences.

Transparent methodology and the use of supervision throughout the analysis sought to reduce subjective bias (France et al., 2019). This was particularly important given that the authors both have previous experience working as HCWs and have experienced client death. As well as transparent methodology, the two reviewers worked together throughout the analysis to consider different possible interpretations. Agreement was reached, however, we may not have covered all the possible ways to interpret the experiences of the home carers involved.

### Conclusion

This review underscores the pressing need for more comprehensive support for the emotional and pragmatic needs of HCWs in the aftermath of a client’s death. Given the context of an ageing global population and potential future care crisis, further research in this area is not just beneficial, but crucial. An increased focus on developing and assessing interventions to support HCWs is welcomed to build a more substantial evidence base. This, in turn, can inform practice recommendations and improve organisational policies and procedures addressing the health and wellbeing of HCWs. HCWs require education and assistance in coping with grief and loss, and organisational culture must change to reflect the impacts of grief and associated support needs.

## Supplemental Material

Supplemental Material - How Do Home Care Workers Experience A Client's Death, Professionally and Personally? A Systematic Review and Meta-Synthesis Supplemental Material for How Do Home Care Workers Experience A Client's Death, Professionally and Personally? A Systematic Review and Meta-Synthesis by Gillian Mhairi Findlay, and Noelle Robertson in OMEGA - Journal of Death and Dying
