# Supplementary material for: How Do Home Care Workers Experience A Client's Death, Professionally and Personally? A Systematic Review and Meta-Synthesis
Source: Omega (Westport). 2024 Nov 28;94(1):189–214. doi: 10.1177/00302228241302431 (PMC13646400; doi:10.1177/00302228241302431)
Supplement: Supplementary file 1 [file sj-pdf-1-ome-10.1177_00302228241302431.pdf]

SUPPLEMENTARY MATERIAL

CASP Quality Appraisal

The CASP checklist questions (listed below) are designed to support researchers in systematically considering quality issues.

Answer 'Yes' scores one point (depicted as green on the table),

Answer 'No' scores zero points (depicted as red on the table).

The option 'Somewhat' was included to optimise the CASP tool and was assigned half a point (depicted as yellow).

1. Was there a clear statement of the aims of the research?
2. Is a qualitative methodology appropriate?
3. Was the research design appropriate to address the aims of the research?
4. Was the recruitment strategy appropriate to the aims of the research?
5. Was the data collected in a way that addressed the research issue?
6. Has the relationship between researcher and participants been adequately considered?
7. Have ethical issues been taken into consideration?
8. Was the data analysis sufficiently rigorous?
9. Is there a clear statement of findings?
10. How valuable is the research?

Study	CASP Question										Score
	1	2	3	4	5	6	7	8	9	10	
Barooah et al. (2019)*	Green	Green	Green	Green	Green	Red	Yellow	Yellow	Yellow	Green	7.5
Boerner et al. (2016)*	Green	Green	Green	Green	Green	Red	Yellow	Yellow	Yellow	Green	7.5
Gee and Yates (2021)	Green	Green	Green	Green	Green	Yellow	Yellow	Yellow	Green	Green	8.5
Ghesquiere and Bagaajav (2020)	Green	Green	Green	Green	Green	Red	Yellow	Green	Green	Green	8.5
Gleason et al. (2016)*	Green	Green	Green	Green	Yellow	Red	Yellow	Yellow	Yellow	Green	7
Tsui et al. (2019)	Green	Green	Green	Green	Yellow	Yellow	Green	Yellow	Green	Green	8.5
Tsui, La Monica, et al. (2022)**	Green	Green	Green	Green	Green	Yellow	Yellow	Green	Green	Green	9
Tsui, Reckrey, et	Green	Green	Green	Green	Yellow	Yellow	Yellow	Yellow	Green	Green	8

al. (2022)**											

Search term strategy

Search terms utilised for the systematic literature review

Sample	Phenomenon of interest	Design	Evaluation	Research type
**professional care**	bereavement	qualitative*	impact*	Qualitative or mixed methods (applied at screening stage)
**live-in/live in**	grief	interview*	effect*	
**in-home/in home**	mourning	focus group*	experienc*	
**home*care*/home care***	death N4 patient	phenomenolog*	attitude*	
**home*help*/home help**	loss N4 patient	ipa	perspective*	
**home*care aid***	death N4 client	hermeneutic*	view*	
**care in the community/community car***	loss N4 client	ethnograph*		
		narrative*		
**home-based care*/Home based*care***		thematic analysis		
		content analysis		
**home*care work*/home care work***		discourse analysis		
**home health aid*/home*health aid***		grounded theory		
**personal*care aide*/personal care aide***				
**home*care support worker*/home care support worker***				
**community*care*/community care***				

** Terms were truncated to maximise relevant returns.*

Searches completed for all databases on 24/06/24

CINAHL

"professional care*" or "live-in" or "live in" or "in-home" or "in home" or "home*care*" or "home care*" or "home*help*" or "home help" or "home*care aide*" or "home-based care*" or "home based*care*" or "home*care work*" or "home care work*" or "home health aide*" or "home*health aide*" or "personal*care aide*" or "personal care aide*" or "home*care support worker*" or "home care support worker*" or "community*care*" or "community care*"

AND

bereavement or grief or mourning or (death N4 patient) or (loss N4 patient) or (death N4 client) or (loss N4 client)

AND

impact* OR effect* OR experienc* OR attitude* or perspective* or view*

AND

qualitative* or interview* or focus group* or phenomenolog* or ipa or hermeneutic* or ethnograph* or narrative* or "thematic analysis" or "content analysis" or "discourse analysis" or "grounded theory"

APA PsychInfo

"professional care*" or "live-in" or "live in" or "in-home" or "in home" or "home*care*" or "home care*" or "home*help*" or "home help" or "home*care aide*" or "home-based care*" or "home based*care*" or "home*care work*" or "home care work*" or "home health aide*" or "home*health aide*" or "personal*care aide*" or "personal care aide*" or "home*care

support worker*" or "home care support worker*" or "community*care*" or "community care*"

AND

bereavement or grief or mourning or (death N4 patient) or (loss N4 patient) or (death N4 client) or (loss N4 client)

AND

impact* OR effect* OR experienc* OR attitude* or perspective* or view*

AND

qualitative* or interview* or focus group* or phenomenolog* or ipa or hermeneutic* or ethnograph* or narrative* or "thematic analysis" or "content analysis" or "discourse analysis" or "grounded theory"

Web of Science

"professional care*" or "live-in" or "live in" or "in-home" or "in home" or "home*care*" or "home care*" or "home*help*" or "home help" or "home*care aide*" or "home-based care*" or "home based*care*" or "home*care work*" or "home care work*" or "home health aide*" or "home*health aide*" or "personal*care aide*" or "personal care aide*" or "home*care support worker*" or "home care support worker*" or "community*care*" or "community care*"

AND

bereavement or grief or mourning or (death NEAR/4 patient) or (loss NEAR/4 patient) or (death NEAR/4 client) or (loss NEAR/4 client) *As TOPIC search because of limits of NEAR function*

AND

impact* OR effect* OR experienc* OR attitude* or perspective* or view*

AND

qualitative* or interview* or focus group* or phenomenolog* or ipa or hermeneutic* or ethnograph* or narrative* or "thematic analysis" or "content analysis" or "discourse analysis" or "grounded theory"

Medline:

"professional care*" or "live-in" or "live in" or "in-home" or "in home" or "home*care*" or "home care*" or "home*help*" or "home help" or "home*care aide*" or "home-based care*" or "home based*care*" or "home*care work*" or "home care work*" or "home health aide*" or "home*health aide*" or "personal*care aide*" or "personal care aide*" or "home*care support worker*" or "home care support worker*" or "community*care*" or "community care*"

AND

bereavement or grief or mourning or (death ADJ4 patient) or (loss ADJ4 patient) or (death ADJ4 client) or (loss ADJ4 client)

AND

impact* OR effect* OR experienc* OR attitude* or perspective* or view*

AND

qualitative* or interview* or focus group* or phenomenolog* or ipa or hermeneutic* or ethnograph* or narrative* or "thematic analysis" or "content analysis" or "discourse analysis" or "grounded theory"

Scopus

"professional care*" or "live-in" or "live in" or "in-home" or "in home" or "home*care*" or "home care*" or "home*help*" or "home help" or "home*care aide*" or "home-based care*" or

"home based*care*" or "home*care work*" or "home care work*" or "home health aide*" or "home*health aide*" or "personal*care aide*" or "personal care aide*" or "home*care support worker*" or "home care support worker*" or "community*care*" or "community care*"

AND

bereavement or grief or mourning or (death W/4 patient) or (loss W/4 patient) or (death W/4 client) or (loss W/4 client)

AND

impact* OR effect* OR experienc* OR attitude* or perspective* or view*

AND

qualitative* or interview* or focus group* or phenomenolog* or ipa or hermeneutic* or ethnograph* or narrative* or "thematic analysis" or "content analysis" or "discourse analysis" or "grounded theory"

Extracts from the meta synthesis process

1.Initial Themes

Barooah et al 2019 Immediate aftermath.pdf

1 Notification of death
2 Reassignment to new client
3 Need for time-off
4 'inherent tension in balancing financial security and emotional health'

Boerner 2016 Home health aides experience role of employer policy.pdf

1 contact with client’s family post-death
2 Identified support needs/preferences

Gee et al 2021 Lessons on life and death.pdf

1 Possibility for growth and sense of meaning
2 Personal impact of loss
3 links to own experiences of death
4 coping strategies
5 Boundary setting
6 grieving alone in personal time
7 Lack of support from agencies
8 Identified support needs/preferences
9 Guilt
10 Intensity & duration of relationship with client affects experience
11 personal strength
12 providing good quality care
13 Seeking support elsewhere
14 Support depends on relationship with supervisor
15 contact with clients family post-death

Ghesquire et al 2020 We-take-care-of-people....pdf

1 Identified support needs/preferences
2 contact with clients family post-death
3 coping strategies
4 'inherent tension in balancing financial security and emotional health'
5 Intensity & duration of relationship with client affects experience
6 links to own experiences of death
7 Need for time-off

8 Lack of support from agencies
9 Notification of death
10 Reassignment to new client
11 Religious supports
12 Seeking support elsewhere
13 Support from colleagues
14 Grief not recognised
15 grieving alone in personal time
16 Emotional reactions

Gleason et al 2016 Supporting home health aides through a client's death role of supervisors.pdf

1 Identified support needs/preferences
2 Notification of death
3 providing good quality care
4 Grief not recognised
5 contact with clients family post-death
6 'inherent tension in balancing financial security and emotional health'
7 Lack of support from agencies
8 support requested

Tsui-et-al-2019-home-care-workers-experiences-of-client-death-and-disenfranchised-grief.pdf

1 Identified support needs/preferences
2 Impact of agency policy/expectations
3 'inherent tension in balancing financial security and emotional health'
4 Intensity & duration of relationship with client affects experience
5 Lack of support from agencies
6 Emotional reactions
7 grieving alone in personal time
8 Grief not recognised
9 Financial impact
10 Boundary setting
11 coping strategies
12 personal strength
13 Possibility for growth and sense of meaning
14 Need for time-off
15 Recognition from family members
16 Seeking support elsewhere
17 Work as a distraction technique

tsui- reckrey et-al-2022-awareness-acceptance-avoidance-home-care-aides-approaches-to-death-and-end-of-life-care.pdf

1 contact with clients family post-death
2 Experience with EOL care
3 Emotional reactions
4 Job based support
5 personal strength
6 Whether death expected

Tsui-LaMonica-et al 2022 Expanding conceptualization of support in low-wage carework.pdf

1 Identified support needs/preferences
2 Family supports
3 Job based support
4 Religious supports
5 self-support
6 Financial impact
7 'inherent tension in balancing financial security and emotional health'
8 Lack of support from agencies
8 Need for time-off
9 contact with clients family post-death
10 coping strategies
11 Support from colleagues
12 Union support

2. Example of reducing themes into relevant categories and translating studies

**Category: Disenfranchised grief**	**Translating metaphors between studies**	**Similarities/differences**
Contact with client's family post-death<br><br>Tsui-LaMonica-et al 2022<br>Tsui- reckrey et-al-2022<br>Boerner 2016<br>Gee et al 2021<br>Ghesquire et al 2020<br>Gleason et al 2016<br>Tsui-et-al-2019	Findings from Tsui, LaMonica et al. (2022) show that contact with client's families was important for recognition of their grief and reciprocal emotional support – particularly around collective grieving rituals. However, this was often against agency policy which precluded ongoing contact with the family.<br><br>Tsui, Reckrey at al (2022) also reported providing support to family members to honour the client they had lost.<br><br>Boerner (2016) agreed on the points that HCWs preferred contact with clients' families and to participate in collective grief rituals but found policies restrictive in relation to this and so often went against these.<br><br>Gee et al (2021) also highlighted HCWs' preference for contact and the importance of ongoing contact for memories.	Similarities:<br><br>- Preferences for contact,<br>- against agency policies,<br>- HCWs went against such policies,<br>- way of engaging in grief rituals,<br>- way to honour and remember client.<br>- Supports individual coping.<br><br>Differences:<br><br>- Contact (although preferred) could be hard to manage at times re boundaries.

	The findings from Ghesquire et al. (2020) also focus on the importance of ongoing contact in an HCW's grief experiences and the lack of recognition of this in agency policy but further, explore the challenges around this ongoing contact and managing boundaries appropriately. Ghesquire et al (2020) and Geason et al (216) explored how contact could support coping and offer a sense of connection for HCWs.<br><br>Tsui et al 2019 added to the theme around the role of family members in promoting recognition of the HCWs role by making contact with agencies.	
- Grief not recognised<br><br>Tsui-et-al-2019<br>Ghesquire et al 2020<br>Gleason et al 2016	Tsui et al (2019) highlighted that HCWs are seen as having insufficient losses and insufficient relationships to justify grief or merit support. Relationships are not recognised and therefore neither is the loss. This may be in part due to agencies discouraging relationships (and therefore not willing to recognise them at death).<br><br>Ghesquire et al (2020) also found grief was present but rarely acknowledged. This disenfranchisement of grief could impact on HCWs out of work as well as increase likelihood	Similarities:<br><br>- Grief not recognised/acknowledged,<br>- Impact on wellbeing in work and in personal lives

	of burnout and compassion fatigue.<br><br>Gleason et al (2016) described the importance of honouring the client as a human being and not just a case number.	
- grieving alone in personal time<br><br>Tsui-et-al-2019<br>Ghesquire et al 2020<br>Gee et al 2020	Tsui et al. (2019) described the expressions of personal grief outwith work and, for some HCWs, an absence of support or understanding in their personal lives.<br><br>Gee et al. (2020) spoke of compartmentalising grief and only letting it sink in once home from work, leading to outpourings of grief for some that impacted sleep.<br><br>Ghesquire et al. (2020) highlighted that grief for clients could trigger grief for previous losses and the need to set these feelings aside in order to work.	Similarities:<br><br>- Compartmentalising grief so can work
- Impact of agency policy/expectations<br><br>Tsui-et-al-2019<br>Tsui-LaMonica-et al 2022	Tsui et al.'s (2019) findings were around HCWs developing strategies based on awareness of subtle agency-driven grieving rules regarding how they should think and feel – often based on policies prohibiting relationships and not getting close. Agencies, therefore, did not recognise grief and could blame HCWs for getting too	Similarities:<br><br>- Agency rules prohibiting relationships,<br>- Blame if HCWs are grieving (as against the relationship rule),

	close. However, some agencies bend the rules if HCWs have worked with clients a long time and allow attendance at funerals. In other cases, HCWs would not always know when funerals were happening, even if they wished to attend.<br><br>Tsui, La Monical et al. (2022) highlighted agencies' reliance on HCWs having their own personal support and not offering any agency support. They also documented the tendency for agency policies to discourage relationships and how policies can prevent HCWs from accessing meaningful emotional support.	- HCWs must rely on personal support, but this is not always present or sufficient.<br><br>Differences:<br><br>- Some agencies bent rules and permitted funeral attendance based on the length of the relationship.
- links to own experiences of death<br><br>Gee et al 2021<br>Ghesquire et al 2020	Gee et al.'s (2021) findings highlight the dual grief experiences HCWs may be subject to as their current grief for the client triggers emotions and memories of previous personal losses.<br><br>Ghesquire et al (2020) also spoke to HCWs experiencing personal grief, and this could have positive and negative impacts - it could increase compassion but evoke strong emotions for the HCW.	Similarities:<br><br>- Grief for the client can bring up strong emotions from previous losses,<br>- Can increase compassion

- Need for time-off<br><br>Tsui-et-al-2019<br>Tsui-LaMonica-et al 2022<br>Barooah et al 2019<br>Ghesquire et al 2020	Tsui et al. (2019) reported findings of a conflict between HCWs wishing to take time-off to grieve but experiencing financial difficulty due to loss of wages if they did take time-off.<br><br>Tsue, LaMonica et al. (2020) also explored HCWs' need for time-off alongside their difficulty in recognising any agency's responsibility to support them in this regard – instead of seeing it as their choice to go off.<br><br>Barooah et al. (2019) highlighted that a lack of time between clients could feel like a lack of recognition that they are human and need time to recuperate from the experience.<br><br>Ghesquire et al. (2020) also reported findings that HCWs would like time-off following a client's death.	Similarities:<br><br>Desire to take time-off if needed,<br>Lack of recognition of emotional impact of work,<br>Not being treated as human
- Notification of death<br><br>Barooah et al 2019<br>Ghesquire et al 2020<br>Gleason et al 2016	Barooah et al. (2019) emphasised the link between HCWs' preferences and agency actions in reassignment. There needed to be a conversation with the HCW to identify what would best support that HCW following the client's death. Some HCWs preferred quick reallocation for financial or emotional reasons (distraction from grief). Having an individual	Similarities:<br><br>Desire for more considerate notification f death, linked to recognition of impact and enfranchising grief.<br><br>Differences:

	approach supports enfranchising the HCW's grief.<br><br>Ghesquire et al. (2020) found that HCWs were not always notified of the death, and when they were, it was not always done considerately.<br><br>Gleason et al. (2016) also spoke of the impact on HCWs of the way in which they were notified and how this related to grief experiences.	Emphasis on an individualised approach to time-off as some may prefer to keep working.
- Personal impact of loss<br><br>Gee et al 2021	Gee et al (2021) found that HCWs experienced multiple emotional impacts of the loss but would often suppress these to continue doing the job.	

3. Data extraction excerpt – example of first and second-order constructs.

Source	First order constructs (participant quotes)	Second order constructs (author comments and interpretation)	Developing third order constructs
**Barooah et al (2019)**	"Well, in a way I took my mourning days although the agency does not pay. The co-ordinator called me for a new case, but I told her I was not prepared for a new case and I had three days with a headache. They respected it. I could not work. I was everything for [client]. I had to take my mourning time. After a week I had to come back because of the bills." (Female HHA, age 52, 72 months with client)<br><br>"After that, I didn't have a permanent one. I was taken from one place to another. [Probe: "How was that for you?"] It wasn't okay, but I needed a job so I had to do it." (Female HHA, age 25, 18 months with client)<br><br>"The patient didn't die well, and an hour after she died, they were giving me another case! They treat me like I was a number, that's it-not a human being. I didn't get much time to rest only a day or 2. They should give you a little time to kind of recuperate." (Female HHA, age 46, 9 months with client)	One-third of the HHAs did not want to be quickly reassigned. They reported negative experiences if they were not given time to recuperate from the previous client's death and positive experiences when they felt their agency respected their need to take time-off. HHAs often share a close relationship with the clients and some took the time-off to grieve and prepare themselves for a new client.<br><br>A little over 21% of the HHAs complained about not getting a permanent reassignment after their client's death, noting they were unhappy because<br><br>they were moved from one client to another and needed more hours.<br><br>Others who did not have that opportunity to take time-off, felt disrespected.	Needing time-off to grieve/prepare for a new client but having bills to pay necessitating work. Taking days as sick days for headache.<br><br>Losing income, not having as satisfactory working conditions after client death - lack of choice.<br><br>Needing time-off but no provision for this. Treated like a number.<br><br>Not being told about death.<br><br>Lack of recognition of the emotional impact this would have.<br><br>Positive experience when aides are notified (even outwith obligation to).

	"It was a little heartless. I know this is our profession, but she was a living human being. They could have been a little more sympathetic. I was with her for two weeks, 4 hours every day, tried to make her feel comfortable. You start to feel for this person. 'Don't go back, she passed'- a little heartless. [Probe: How could they have said it differently?] Maybe a few words could have been a little different. It was like saying, 'The supermarket doesn't have this, go to the next one.'" (Female HHA, age 33, 5 months with client)	There were different types of notification pathways through which HHAs learned about their client's death. Only 42.5% reported being notified, and of this group, 22.5% were notified by their agency/coworkers and 20% by their client's family/friends. Among the HHAs who were notified by the agency, there were mixed responses about how notification was handled. Some had a negative experience and expressed wanting their supervisor to handle the task of notification more sensitively. Others noted that agencies are not obligated to inform their HHAs about client death due to confidentiality issues, yet they were thankful and had a positive experience when their agency made an effort to notify them.	

**Tsui, Franzosa, et al. (2022)**	"I'm not able to take off any time, because of the money that I earn is not enough to have, to even do anything beyond basically basic needs" (Participant 217, 2 years).<br><br>"No, I didn't get support from [the agency]. … I just took my days off. I chose to lose the pay, right. Because I just—because death is real and it's fragile. Right? And you need a moment to hit the reset" (Participant 215, 3 years).<br><br>"I felt really sad. I don't know if they [the agency staff] know, but I immediately called the cousin and said I'm so sorry about what happened and that she was a really good person. … And her cousin was like, "Oh, she spoke so highly about you. She loved you" (Participant 101, 1 year).<br><br>"The family had a little get together and they invited us [the aides], so we were there sharing her spirit and talking about her. So that was a thing in terms of coping, that little get together" (Participant 216, 8 years).<br><br>"everybody moves on with their lives" (Participant 103, 17 years),<br><br>" . . . you can talk and we laugh about the things that she [the client] used to say. I think if I hadn't been in touch with them, it would've felt sad and lost. … I would've taken it on more" (Participant 202, 20 years).	Aides might want to take time-off after client loss but are typically unable to do so, due to financial pressures. When aides do take time-off, they often see it as their choice to go unpaid, despite the need for time-off stemming from the work.<br><br>Aides sometimes spoke about meaningful experiences of reciprocal emotional support between themselves and family members or friends of the deceased client. Our data indicate that aides might contact a client's family after client death (or vice versa) and find support through shared grief, revisiting memories, and ongoing social interactions. Sometimes this was through a one-on-one interaction.<br><br>Other aides talked about more collective experiences [with families of clients]<br><br>In many cases, this support was short-term as "everybody moves on with their lives" (Participant 103, 17 years), though we also heard about aides who were in touch with the deceased client's families in a more ongoing way. For instance, one aide developed a particularly close relationship with her deceased client's friends. She said, " . . . you can talk and we laugh about the things that she [the client] used to say. I think if I hadn't been in touch with them, it would've felt sad and lost. … I would've taken it on more" (Participant 202, 20 years). It had been a difficult case over three years, with multiple hospital stays interspersed among periods of home care. Throughout this time, this aide had worked together with the client's neighbors and ex-husband to provide care and stay in touch. After the client's death, they checked in on each other, spent	Needing time-off but being on a low wage means no scope to lose any income from absence.<br><br>Lack of support. Unable to take time-off due to financial implications. See it as a personal issue rather than work-based issue.<br><br>Mutual support with family members of clients<br><br>Contact with family members may be short term or longer term depending on relationships.

4. Data extraction excerpt – example of finalising the third-order constructs.

Tsui-et-al-2019-home-care-workers-experiences-of-client-death-and-disenfranchised-grief.pdf	Gee et al 2021 Lessons on life and death.pdf	Ghesquire et al 2020 We-take-care-of-people....pdf	Interpretation

Our focus groups and peer interviews suggest that the nature of this relationship (especially when the client is “good to you” [FG2]) powerfully shapes an aide’s experience of death.<br><br>A majority of the relationships described in our data appeared to be intimate and socially nourishing, which understandably increased the perception of loss when clients passed away.<br><br>However, participants acknowledged that not all relationships were positive and that could affect the impact of a client’s death. For instance, one participant whose client was “never pleased” and “calling you all kind of name” described her response to this client’s death as less emotional compared with other clients (FG).	All carers treated caring as more than a job, not only to provide the best quality of care but also for their themselves, “If it doesn’t come from inside, you will not enjoy it” (Rosa). Such investment also increased carers’ vulnerability to grief. Zhita recalled the loss she felt after a patient she had cared for “just three weeks”: “If you put your heart in what you are doing, you will feel it”	Many noted that because they spent so much time with the patient (often working with them 4 to 8 hours a day, every day), they had a closer relationship than with the patient than did other hospice providers. As one HHA described: I find when people are at death’s door, that’s when they’re the most honest, and the most vulnerable, and they’re telling you about their lives and sometimes their mistakes and their regrets. And these are things that I don’t think you can speak to anybody else about, because they told you that in some sort of confidence. This close connection could make the loss particularly painful, and many HHAs described feeling strong feelings of grief when patients died.	The intensity & duration of the relationship with the client affects the experience. Working intensely with clients over a short period of time may have as much impact as working with patients over a longer time period.
As one participant said, “Relationships grow by spending time with the person. Spiritually, there is something going on between you, so you feel the pain of the person. You feel the hate of the person. You feel the joy of the person” (FG).<br><br>And the length of the relationship need not necessarily stretch into years to be considered sufficient to create a sense of loss. As one participant said, “Six months can be long term. It depends on the kind of relationship that you have with your client. [. . .] And if you are an hospice aide that works with a client to give them comfort care to see them go and they’re gone, you’re going to miss the person, too” (FG).		Our focus groups and interviews with HHAs indicated that they have a unique role on the hospice team, characterized by relationships with patients and family members that can be very close, but which are also limited by time and other<br>responsibilities.	

Though agency policies sometimes limited aides' contact with clients' families after a death, one participant talked about a circumstance in which workers might be able to attend a client's funeral (though several noted this would not be considered paid time):You have to be with the client for a long time. And the client is talking about you, you know, how much you help and how much you do this, so understand that there's a kind of relationship between you and the client. Then they [the agency] would say, "Would you like to go to the funeral?" (PI)			
